# Effects of sanitation practices on adverse pregnancy outcomes in India: a conducive finding from recent Indian demographic health survey

**DOI:** 10.1186/s12884-019-2528-8

**Published:** 2019-10-24

**Authors:** Ratna Patel, Ajay Gupta, Shekhar Chauhan, Dhananjay W. Bansod

**Affiliations:** International Institute for Population Sciences, Govandi station Road, Deonar, Mumbai 400088 India

**Keywords:** Adverse pregnancy outcome, Sanitation, Low birth weight, India, National family health survey, Access to toilet

## Abstract

**Background:**

Several risk factors predisposing women and their live-borns to adverse outcomes during pregnancy have been documented. Little is known about sanitation being a factor contributing to adverse pregnancy outcomes in India. The role of sanitation in adverse pregnancy outcomes remains largely unexplored in the Indian context. This study is an attempt to bring the focus on sanitation as a factor in adverse pregnancy outcome. Along with the sanitation factors, few confounder variables have also been studied in order to understand the adverse pregnancy outcomes.

**Methods:**

The study is based on the fourth round of National Family Health Survey (NFHS-IV) covering 26,972 married women in the age-group 15–49. The study variables include the mother’s age, Body Mass Index (BMI), education, anemia, and Antenatal care (ANC) visits during their last pregnancy. The household level variable includes place of residence, religion, caste, wealth index, access to toilet, type of toilet, availability of water within toilet premises, and facility of hand wash near the toilet. Children study variables include Low Birth Weight (LBW), the order of birth (Parity), and the death of the children of the women in the last 5 years. The target variable Adverse Pregnancy Outcome (APO) was constructed using children born with low birth weight or died during the last pregnancy.

**Results:**

We calculated both adjusted as well as unadjusted odds ratios for a better understanding of the association between sanitation and adverse pregnancy outcomes. Findings from the study showed that women who did not have access to a toilet within the house had a higher risk of adverse pregnancy outcome. In the multivariable model, no association was observed for adverse pregnancy outcome among women who did not have access to toilet and women who used shared toilet. Teenage (15–19 years), uneducated, underweight and anemic mothers were more likely to face APO as compare to other mothers in similar characteristics group.

**Conclusions:**

Our findings contribute to the decidedly less available literature on maternal sanitation behaviour and adverse pregnancy outcomes. Our results support that sanitation is a very significant aspect for women who are about to deliver a baby as there was an association between sanitation and adverse pregnancy outcome. Education on sanitation practices is the need of the hour as much as it needs to follow.

## Background

The significance of sanitation in the follow up of Adverse Pregnancy Outcomes (APOs), which includes both preterm births and low births weight [1, 2], is widely studied in developed as well as developing countries [3–5]. Around the globe, nearly 20 million infants are born annually with low birth weight [6]. Low birth weight is an important indicator in assessing neonatal health that also comprises babies born preterm (less than 37 completed weeks of gestation). Adverse pregnancy outcomes can occur by any of the four possible ways: when women lose their baby during early pregnancy, i.e., miscarriage or spontaneous abortion, when women lose their baby during late pregnancy, i.e., stillbirths, when women have baby earlier than expected, i.e., preterm birth, or when women have a baby with low birth weight. The World Health Organisation defines a birth weight of less than 2500 g as low birth weight [7].

The causal mechanism of adverse pregnancy outcomes has not been established satisfactorily as there are many reasons for the onset of adverse pregnancy outcomes. Studies have reported numerous risk factors for APOs such as Obesity [8–11], anemia [12–18], diabetes [19–21], antenatal care [22], maternal tobacco consumption [23], history of abortion [24–27], environmental pollution [28–32], hypertension [33, 34] and many others. Exposure to unsafe water, poor sanitation, and poor waste management during the period of pregnancy may increase the risk of infection in the mother and may lead to low birth weight babies and preterm deliveries [35, 36].

Despite persistent efforts, access to sanitation remains limited in many developing countries, including India. Sanitation is one of the crucial factors essential for the health of mother and to be born baby. Various studies link adverse pregnancy outcomes and poor child health to the poor sanitation [5, 37, 38]. One of the Sustainable Development Goals (SDGs) discusses sanitation. SDG-6, ensuring environmental sustainability, sets a target related to water and sanitation where it commits to 50% reduction in the proportion of people without access to safe drinking water and basic sanitation between 1990 and 2015.

Inadequate provision of sanitation facilities is one of the causes of worry in India as sanitation contributes to adverse pregnancy outcome. The major causes and predictors of adverse pregnancy outcomes are not well understood in India because of the considerable variation in the availability and quality of the data that underestimates the actual number of adverse pregnancy outcomes. There is an urgent need to increase the quality of data on pregnancy outcomes. Across the globe, around 3 million babies are stillborn every year- more than 8200 babies a day [39]. With such a large number of just stillbirths, it is horrifying to imagine the total number of adverse pregnancy outcomes. The importance of adverse pregnancy outcomes has risen on the policy agenda in recent times, however, India still has a long way to go. More than 60% of preterm births occur in just South Asia and Sub-Saharan Africa [3]. A study estimated that nearly 12.8 million babies were born with LBW in India in the year 2010; this accounts for 47% of all births [1].

The objectives of this study are two-fold- first to study the association between sanitation factors and adverse pregnancy outcomes among mothers aged 15–49 years. The second is to study the prevalence of sanitation and adverse pregnancy outcomes in India and its states. The study also focuses on various confounder variables that may affect adverse pregnancy outcomes along with the sanitation factors.

### Sanitation in India

The study of sanitation in India goes as back as 1912 when a book was written on the topic [40]. Although a few books or articles had been written earlier, this was a comprehensive treatise on this issue to the author’s best knowledge. Sanitation in India continues to be inadequate, despite longstanding efforts. House-listing and housing census of India, 2011, revealed that as many as 53% of the households do not have drainage facilities within their premises. The Indian government in the year 2014 launched a nation-wide mission known as “Swachh Bharat Mission” (SBM) to accelerate the efforts to achieve universal sanitation coverage. The mission aims to make India clean by the year 2019. Separate ministries look after the Swachh Bharat Mission in rural and urban areas. In rural areas, the Ministry of Drinking Water and Sanitation is entrusted to look after the sanitation progress, while in urban areas, the Ministry of Housing and Urban Affairs does the same. The Ministry of Drinking Water and Sanitation claims that with the inception of Swachh Bharat Mission on October 2, 2014, it has built nearly 80 million toilets, with the number of toilets increasing every day.

### Adverse pregnancy outcomes in India

There are very few studies that have directly discussed the effect of sanitation on adverse pregnancy outcomes in India [5]. A survey on a cohort of women from urban slums in New Delhi, Kolkata, and Chennai and rural slums in Hyderabad, Varanasi, and Chandigarh in 1981 recommended that improving sanitation and hygiene would have a positive effect on lowering the adverse pregnancy outcomes including prenatal and neonatal mortality [41].

Although there were a few studies in the Indian context relating the adverse pregnancy outcomes to causes other than sanitation, linking to sanitation are very limited. In a survey undertaken in the Indian state, West Bengal, it was found that exposure to a high concentration of arsenic during pregnancy was associated with a six-fold increased risk of stillbirth [42]. Another study identified different socio-economic determinants of low birth weight by following a community based prospective cohort in 45 villages of Pune district in India [43].

## Methods

### Data source

The fourth round of National Family Health Survey (NFHS), also known as Demographic Health Survey (DHS) - India, conducted in 2015–16, was utilized for this study. NFHS is one of the biggest population-based surveys in India which collects information on health and nutrition for each Indian state and union territory. The survey was designed to provide estimates of the prevalence of anemia, HIV, glucose level, and malnutrition. The sampling frame for NFHS-4 was the Census of India 2011. Census Enumeration Blocks (CEBs) and Villages in urban and rural area respectively served as Primary Sampling Unit (PSUs). Two-stage stratified random sampling was used to get the samples separately in urban and rural areas in each of the districts. In rural areas, stratum was a part of approximately six equal substrata, created on the basis of the estimated number of households in each village, and also the percentage of the population belonging to schedule caste and scheduled tribe (SC/ST).

Within each explicit rural sampling stratum, PSUs were sorted according to women literacy rate before PSU selection. All PSUs were selected with probability proportionate to the size of the PSU within each stratum in the first stage. The house listing was done in all selected PSUs, which served as a sampling frame for household selection. If the selected PSU had 300 or more households, then they were segmented into 100–150 households, out of which one was selected through the PPS method. A fixed number of 22 households per cluster/PSU was selected during the second stage. Similarly, CEBs and households were selected in urban areas. Around 628,900 households were selected initially for the survey, out of which 616,346 were occupied. Among the occupied, 601,509 were interviewed successfully with a response rate of 98%. Among the interviewed households, 699,686 women aged 15–49 completed the women’s interview while 112,122 men aged 15–54 completed interview with a response rate of 97% and 92%, respectively. It was noted that men were selected for interview only for the state module that provided state-level estimates, while women’s indicators were analyzed at the district level.

Anthropometry measures including height and weight were measured for children using a standard instrument that included SECA 417 infantometer (for the height of children under 2 years), SECA 213 stadiometer (for the height of children under 24–59 months and adults) and SECA 874 (for weight of other children and adults) in this survey. Health investigators collected blood specimens for anemia from eligible women aged 15–49 years, children aged 6–59 months and men aged 15–54 years. For this, prior consent was taken from them and their parents (for children under 6–59 months). Blood sample was taken from the fingertip, collected in a micro cuvette, and hemoglobin analysis was done. A hemoglobin level below 9 g/deciliter (g/dl) among pregnant women and 7 g/dl among non-pregnant women/men and children was considered as severely anemic (NFHS Report, 2015–16).

The sample of the current study included only the married women who gave birth in the past 5 years and their last born child. The total sample of the study includes 26,972 women aged 15–49 years.

## Measures

### Independent variable

The study variables included the mother’s age, body mass index (BMI), education, anemia, and ANC visits during their last pregnancy. The household-level variables included the place of residence, religion, caste, wealth index, access to the toilet, type of toilet, availability of water within toilet premises, and facility of hand wash near the toilet. Study variables for children included low birth weight (LBW), the order of birth (Parity), and death of children of women occurring in the last 5 years. All the independent variables were included in this study after going through the literature and discussion in the introduction. In the Indian context, religion, caste, and place of residence play an important role. It is worth stating that Southern states are more developed as compare to other parts of India.

Mother’s age, education, and anemia level have been given in the data, while BMI was calculated using the women’s weight (in Kg) divided by height (in meter) square (BMI = weight / height2). The BMI for pregnant women was not calculated and marked as missing for the current study (11% of women were pregnant at the time of the survey). BMI is categorized by the World Health Organization (WHO) for South-East Asian countries as underweight (BMI < 18.5), Normal (18.5–25.0) and Overweight/obese (BMI > 25.0). This study used the same categorization. The number of ANC visits has been given in the data and categorized as 0, 1, 2, and 3+ as standard WHO rules, i.e., minimum of three ANC visits should be made during the pregnancy. Characteristics of children such as LBW and parity were determined while the variable related to death of a child was directly available from the data. LBW was determined using the birth weight of the child as given in the data wherein children weigh less than 2500 g were considered as LBW. Parity was determined using the data available as total number of children ever born to a woman.

Wealth index for household (Poorest to Richest), access to toilet, toilet type, availability of water and hand wash near toilet, religion (Hindu, Muslim, and others) caste (Schedule caste-SC, Schedule Tribe- ST, Other Backward Caste-OBC, and others) of household were directly available from the data. The wealth index was based on the number and kinds of consumer goods, the household owned.

### Dependent variable

The target variable Adverse Pregnancy Outcome (APO) was determined using children who were born with low birth weight or died during the last pregnancy. APO among mother represents that either the child born with low weight or died. The NFHS data only give LBW and death occurred to a woman for her last pregnancy, so we could only add up these two variables in the formation of the variable of adverse pregnancy outcome.

### Analysis

The data was analyzed using bivariate (Chi-square for finding the association) and multivariate techniques (Logistic regression) in SPSS v25. Only those variables were considered in the logistic models, which were found to be associated. Adjusted odds, as well as unadjusted odds, were calculated to measure the difference between the target variable. Appropriate weights were applied to account for the intricate sampling design and respondent attrition as available in the data. To ensure the actual represention of the survey results at the national as well as at the domain level, sampling weights were required. Since the survey has utilized the two-stage sampling in the rural areas and three-stage sampling in the urban areas; weights were calculated using the sampling probabilities separately for each sampling stage. The final weights were normalized to give a total number of weighted cases that equals to the total number of un-weighted cases at the national level. These weights are used in this study to analyze the data and make it generalized. ArcGIS software is also used for generating maps showing the prevalence of adverse pregnancy outcomes and the percentage of households with no access to toilet in India.

## Results

### Sample characteristics

Table 1 presents the sample characteristics. Nearly 70% of the women were in the age group 20–29. One in every four women was underweight while 14% were overweight. 38% of the women belonged to the first parity, 33% to second parity, while remaining women belonged to 3 or more parity. Nearly one in every ten women was bereft of any education, while only one in every ten women had higher education. Almost 16% of the women were severely or moderately anemic, while 40% of women were mildly anemic. Around 34% of the women had less than the required (at least three ANC visit) ANC visit, while almost 15% of women had no ANC visit. Almost 40% of the women did not have any access to a toilet, while 16% of the women used a shared toilet.

**Table 1:** Percentage distribution of the selected sample of women aged 15–49 years and Bivariate distribution of adverse pregnancy outcome (APO) by women’s selected socio-demographic, anthropometrics, and clinical characteristics

Sample characteristics	Sample percentage	Population (Total)	Percentageof APO
Age		**(26,972)**	
15–19	4.17	1125	23.18
20–24	33.68	9084	19.78
25–29	35.63	9611	17.25
30–34	17.57	4739	17.53
35–39	6.8	1834	18.51
40–44	2.15	579	18.81
BMI		**(23909)**	
Underweight	27.89	6668	21.99
Normal	57.91	13,846	17.39
Overweight	14.2	3395	15.33
Parity		**(26,972)**	
1	37.66	10,158	19.27
2	32.53	8773	17.32
3 +	29.81	8041	18.82
Education		**(26,972)**	
No Education	25.96	7002	20.73
Primary	14.87	4011	20.75
Secondary	49.39	13,322	18.12
Higher	9.78	2637	14
Anemia		**(26482)**	
Severe	1.18	319	26.43
Moderate	14.76	3981	20.16
Mild	39.63	10,688	18.02
Not Anaemic	42.61	11,494	18.12
Wealth Index		**(26,972)**	
Poorest	21.95	5920	20.8
Poorer	22.71	6124	19.67
Middle	21.04	5675	18.73
Richer	19.19	5176	18.36
Richest	15.12	4077	14.72
Place Of Residence		**(26,972)**	
Urban	27	7282	17.46
Rural	73	19,690	18.92
ANC Visit		**(26,972)**	
0	14.64	3948	21.28
1	5.49	1480	19.71
2	13.63	3677	19.83
3+	66.24	17,867	17.65
Religion		**(26,972)**	
Hindu	78.79	21,252	18.74
Muslim	13.69	3692	17.4
Others	7.52	2028	16.77
Caste		**(26,972)**	
SC	20.75	5597	19.52
ST	15.75	4249	20.66
OBC	40.39	10,894	18.08
Others	23.11	6232	17.38
Access To Toilet		**(26,972)**	
No Access	40.47	10,916	20.01
In House	43.2	11,652	16.72
Shared Toilet	16.33	4404	19.79
Type Of Toilet		**(26,972)**	
No Toilet	40.47	10,916	20.01
Flush Toilet	43.82	11,818	17.21
Pit Latrine	8.33	2246	17.5
Other	7.39	1992	19.75
Availability Of Water		**(26,972)**	
No Availability	18.82	5075	20.47
Available	81.18	21,897	18.05
Availability Of Hand Wash		**(26,972)**	
No Availability	40.18	10,837	19.32
Available	59.82	16,135	17.9

Bold entries are significant values

### Adverse pregnancy outcomes and no access to toilets in Indian states

**Fig. 1:**
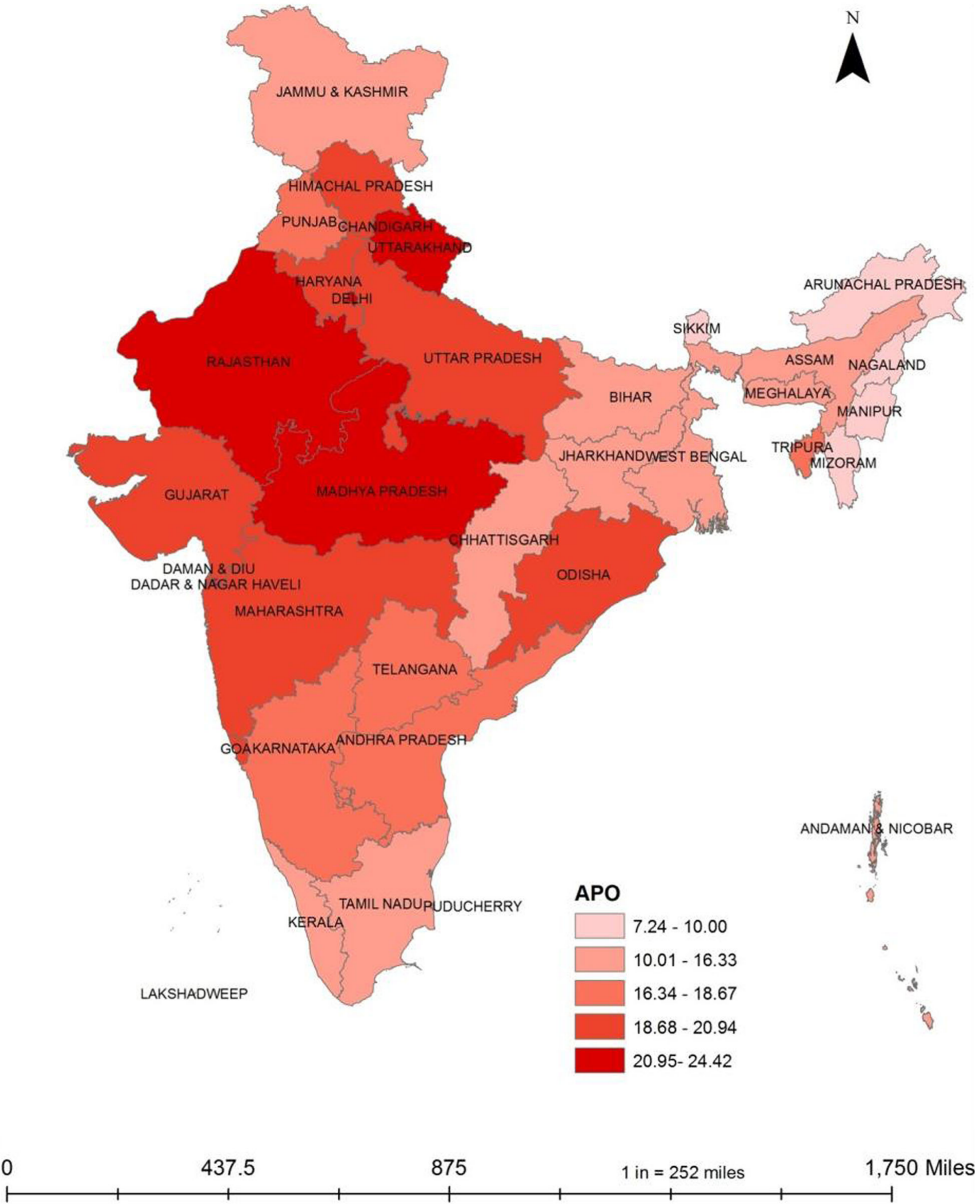
Prevalence of Adverse Pregnancy Outcome (APO) in India, NFHS-4, 2015–16. Data Source:- Authors created MAP using NFHS 4 data in Arc-GIS 10

Figure 1 shows the prevalence of Adverse Preganancy Outcomes in India. The APOs are more prevalent in North India and least pervasive in North-Eastern states of India. In states like Uttarakhand, Madhya Pradesh, Rajasthan, Odisha, Uttar Pradesh, Haryana, and Goa, one in every five pregnancy turned out to be adverse. The national prevalence of 18.44 was better than ten states of the country.

**Fig. 2:**
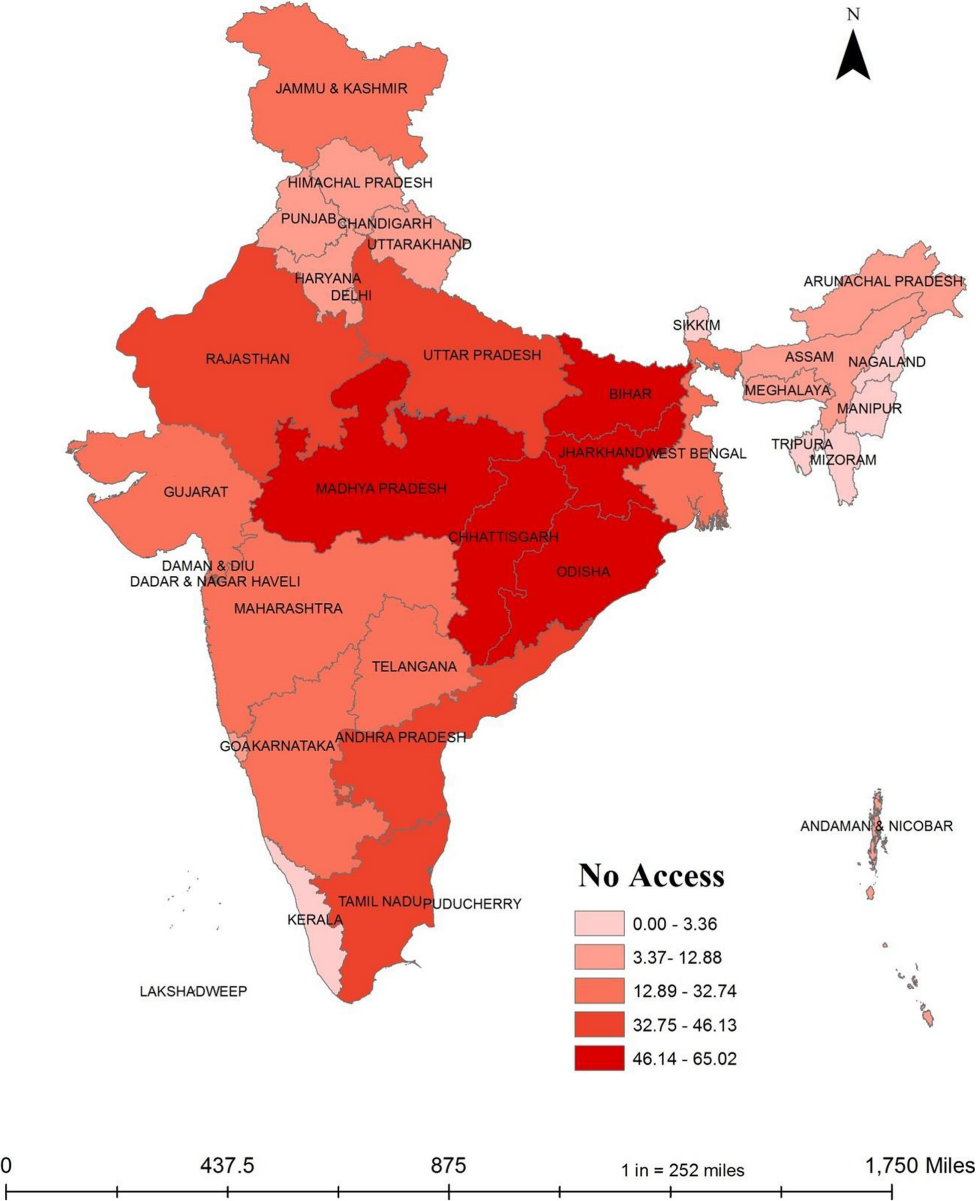
Prevalence of No-Access to toilet facility in India, NFHS-4, 2015–16. Data Source:- Authors created MAP using NFHS 4 data in Arc-GIS 10

Figure 2 shows the percentage of households having access to toilet facility in India. More than half of the households in Jharkhand, Odisha, Bihar, Chhattisgarh, and Madhya Pradesh did not have access to toilet facility, either within household premises or on shared basis. In India, nearly 37% of households did not have access to the toilet.

## Bivariate associations

Table 1 also represents bivariate associations between APO and selected study variables. Twenty-three percent of mothers in teen ages had adverse pregnancy outcomes while this was less among mothers in the 25–34 years age group. Nearly 22% of the underweight mothers had APO during their last pregnancy which was high compared to mothers with normal weight. Among women who gave birth to their first born, 19% of the mothers went through the adverse outcome of pregnancy. Nearly 21% of the illiterate and primary educated mothers had either lost their child or had a child born with low birth weight. The percentage of adverse pregnancy outcomes decreased 6 points from 21% among the poorest mothers to 15% among the richest mothers. The chances of APO decreased as the number of ANC visits made during the latest pregnancy increased from zero to three or more.

### Sanitation and adverse pregnancy outcomes

Nearly 20% of the women who either did not have toilet facilities within the household or were using shared toilets had gone through adverse pregnancy outcomes. Similarly, the adverse consequences of pregnancy were found to be higher among mothers who did not have availability of water and hand wash facility near the toilet.

## Multivariate analysis

Table 2 depicts the unadjusted odds for adverse pregnancy outcomes keeping sanitation factors as independent variables. The mothers who had access to toilet had significantly lower chances of APO as compare to mothers who did not have access to toilets. Similarly, women who had used the flush or pit latrine toilets had significantly lower chances for adverse effects on pregnancy outcome compared with mothers who did not used toilets. Undesirable outcomes have been found to be significantly higher among mothers who did not have the availability of water and hand wash near the toilet.

**Table 2:** Pregnant women’s sanitation access and use with unadjusted odds ratios for adverse pregnancy outcomes

Characteristics	Odds ratio	95% Confidence interval	
		Lower limit	Upper limit
Access to toilet			
No access®	1		
In house	0.77***	0.748	0.792
Shared toilet	0.93***	0.899	0.972
Type of Toilet			
No toilet®	1		
Flush toilet	0.80***	0.776	0.822
Pit latrine	0.75***	0.713	0.787
Other	0.97	0.921	1.024
Availability of water			
No availability®	1		
Available	0.87***	0.842	0.901
Availability of hand wash			
No availability®	1		
Available	0.88***	0.861	0.908

***p < 0.01, **p < .05, *p < 0.1

Table 3 shows the adjusted odds for APO taking sanitation factors as independent factors. There are significantly less chances (0.78, *p*-value< 0.01) of adverse pregnancy outcomes among mothers having toilets in-house as compared with mothers not having access to a toilet. There were also higher chances of APO among the mothers having unavailability of water near the toilet.

**Table 3:** Pregnant women’s sanitation access and use with adjusted odds ratios for adverse pregnancy outcomes

Characteristics	Odds ratio	95% Confidence interval	
		Lower limit	Upper limit
Access to toilet			
No access®	1		
In house	0.78***	0.762	0.811
Shared toilet	0.95	0.912	0.988
Availability of water			
No availability®	1		
Available	0.94**	0.909	0.981
Availability of hand wash			
No availability®	1		
Available	0.98	0.948	1.009

***p < 0.01, **p < .05, *p < 0.1

Table 4 presents the adjusted odds explaining the association between the adverse pregnancy outcome and background characteristics, including sanitation factors. Teenage (15–19 years), uneducated, underweight, and anemic mothers are more likely to face APO as compare to other mothers in similar characteristics group.

**Table 4:** Multivariable adjusted models for the association between sanitation characteristics and adverse pregnancy outcomes

Characteristics	Odds ratio	95% Confidence interval	
		Lower limit	Upper limit
Access to toilet			
No access®	1		
In house	0.87***	0.832	0.901
Shared toilet	1.01	0.966	1.058
Availability of hand wash			
No availability®	1		
Available	1.01	0.981	1.048
Age group			
15–19®	1		
20–24	0.87***	0.806	0.942
25–29	0.79***	0.730	0.857
30–34	0.79***	0.729	0.866
35–39	0.81***	0.740	0.896
40–44	0.80***	0.704	0.900
No education®	1		
Primary	0.99	0.948	1.042
Secondary	0.84***	0.811	0.879
Higher	0.69***	0.647	0.733
Parity			
1®	1		
2	0.89***	0.855	0.919
> 3	0.90***	0.862	0.939
BMI			
Underweight®	1		
Normal	0.78***	0.757	0.810
Overweight	0.76***	0.726	0.801
Anemia			
Severe®	1		
Moderate	0.77***	0.668	0.889
Mild	0.69***	0.602	0.795
Not anaemic	0.67****	0.586	0.775
ANC visit			
0®	1		
1	0.91*	0.845	0.976
2	0.91***	0.859	0.958
> 3	0.84***	0.803	0.875

***p < 0.01, **p < .05, *p < 0.1

The mothers who did not have access to toilets and had not visited for ANC had higher chances of adverse outcomes keeping other factors constant. In the multivariable model, the availability of hand wash did not seem to affect adverse pregnancy outcomes.

## Discussion

An attempt was made to understand if there was any association between maternal sanitation behavior and adverse pregnancy outcomes by using DHS data, also known as NFHS (National Family Health Survey), in India. It was observed that sanitation was an important factor governing adverse pregnancy outcomes. Other causes, besides sanitation, were also examined and these causes were further associated with adverse pregnancy outcomes. Findings contributed to the scant literature on maternal sanitation behavior and adverse pregnancy outcomes. First, results support that sanitation is a very significant aspect for those women who are about to deliver a baby as findings suggest an association between sanitation and adverse pregnancy outcome. Secondly, our findings indicated, in line with other studies, that adverse pregnancy outcomes were also related to socio-demographic characteristics of the mother. This study is important in the Indian context because previous studies/literature/reports (except a study by Padhi et al.; 2015) linking adverse pregnancy outcomes to sanitation were scarce.

### Adverse pregnancy outcomes and sanitation

We calculated both adjusted as well as unadjusted odds ratios for a better understanding of the association between sanitation and adverse pregnancy outcomes. Results showed that women who did not have access to a toilet within the house had a higher risk of adverse pregnancy outcome. In the multivariable model, no association was detected for adverse pregnancy outcomes among women who did not have access to a toilet and women who used shared toilets. Women who neither had access to a toilet at home nor shared a toilet were assumed to practice open defecation. Open defecation is still a problem by and large, and it occurs not only as a result of the unavailability of latrines but also due to cultural and behavioral reasons. There is a scarcity of research linking sanitation to adverse pregnancy outcomes in India, and it paves a difficulty in assessing our findings in the context of other similar findings. This study provides an insight into comparisons on adverse pregnancy outcomes for women who followed sanitary practices versus those who did not along with other socio-demographic factors.

States like Odisha, Madhya Pradesh, Uttar Pradesh, and Rajasthan which had inadequate sanitation coverage also had a higher number of adverse pregnancy outcomes. There is a complicated relationship between sanitation and adverse pregnancy outcomes as sanitation alone could not be a cause for adverse pregnancy outcomes. Many other factors such as, socio-demographic factors played an imperative role in the occurrence of an adverse pregnancy outcome. In spite of this, the importance of sanitation in the occurrence of an adverse pregnancy outcome cannot be ruled out completely.

Women were ignorant about sanitation practices and their importance in avoiding adverse pregnancy outcomes. We reiterate, on the basis of our findings, that sanitation is one of the crucial factors that help prevent adverse pregnancy outcomes. The scarcity in the studies associating sanitation to adverse pregnancy outcomes points out the neglected dimension of sanitation in the follow up of adverse pregnancy outcome. Improved sanitation is a basic need, and many women in India are deprived of it.

### Adverse pregnancy outcomes and other associated causes

Some factors strongly influenced adverse pregnancy outcomes. These include age of the mother, the educational level of the mother, parity, BMI of the mother, ANC visits, and a few others. These findings emphasise that pregnancies during adolescence as well as in the later stages of the reproductive period were associated with an increased risk of adverse pregnancy outcomes. Mothers in the age group 20–24 had high chances of adverse pregnancy outcome as compared to mothers in subsequent age-group [44]. Many studies have confirmed that the chances a pregnancy during early ages will lead to adverse pregnancy outcomes are higher [45–47]. The results comprehensively conclude that education is one of the most critical factors in discouraging the outcome of adverse pregnancy. Our study found that education is a crucial determinant of adverse pregnancy outcome in a multivariable model. Few studies suggest that with the increase in mother’s education, the occurrence of adverse pregnancy outcomes decrease [44]. Educational interventions not only have a positive impact on the delivery outcomes but also affect the health of the pregnant woman. Education was a missing link in the lives of Indian women, as nearly one in every four women in the study sample was without any education.

Risks were high among anemic women. Women who were not anemic had fewer chances of having an adverse pregnancy compared to women who were severely anemic. Maternal anemia was associated with an increased risk of preterm births and low birth weight rates [48–51]. Our findings suggest that underweight women also had a higher chances of facing an adverse pregnancy outcome.

Education on sanitation practices is much required as much as it needs to follow. Pregnant women should be imparted knowledge about the need for sanitation during pre and post-pregnancy period.

## Limitations of the study

Although there is more to sanitation than what we could have captured for the analysis due to data constraints- access to the toilet, availability of water for toilet use, type of toilet, and availability of hand wash, but these measures in no way can be misleading. Sanitation also included access to safe drinking water, and this information is missing from this study. Only two important variables, namely; access to toilet and availability of hand wash, have been used to understand the effect of sanitation on adverse pregnancy outcomes along with other socio-demographic factors in a multivariable model. The data only collected information about the availability of toilet in a household, and did not imply that women in the households used that facility as a few of the previous studies [52] have indicated that people preferred open defecation even though they had access to a toilet within their household premises.

Unmarried women were not taken into consideration as the sample of such women were not available in the data who have/had a child. Although this may not affect our results in the Indian context, as nearly all of the women have child after marriage only. So, therefore, our results may be generalized upon the general population. The results focused on the main factors of adverse pregnancy outcomes (APO) such as child death (infant mortality/ stillbirth) and child’s low birth weight, however APO might include maternal death which was not available from the data. The data was also limited to only the death of a child and did not give reasons for the same which could have been analyzed further. The reason for death was not included in the survey because it could have been tragic to the mother or the household to be asked such a question or due to the recall bias of the correct reason of death.

This is a cross-sectional data source, so the cause-effect relationship cannot be addressed. The study may be affected by recall bias also.

## Conclusions

The results found that women with no access to the toilet had a higher chances of adverse pregnancy outcomes as compared to women having access to the toilets. No access to toilets could be assumed as people practicing open defecation. The government of India launched a sanitation drive known as ‘Swachh Bharat Mission’ in 2014, and building new toilets to address the issue of open defecation is the agenda of the mission. There is also a need to bring a change in behavior of people as even after having IHL (Individual Household Latrines) people prefer to go out for defecation. A study based on SQUAT (Sanitation Quality, Use, Access, and Trends) survey undertaken in five states of India mentioned that in India people prefer open defecation, even after having access to latrines [52].

Few other studies reiterate that people are reluctant to adopt latrine use habits and instead continue to defecate in the open due to certain socio-cultural and behavioral factors [53]. In India, open defecation is widely practiced without any attached stigma [54], and this is one of the reasons why people prefer to choose open defecation in spite of having access to latrines [55].

## Data Availability

The study utilises secondary source of data which is freely available in public domain through http://iipsindia.org. The necessary ethical approval has been taken by the respective organisations involved in the data collection process.

## Abbreviations

ANC: Ante Natal Care
APO: Adverse Pregnancy Outcomes
BMI: Body Mass Index
DHS: Demographic Health Survey
HIV: Human Immunodeficiency Virus
IEC: Information, Education, and Communication
NFHS: National Family Health Survey
SBM: Swachh Bharat Mission
SDG: Sustainable Development Goals
SQUAT: Sanitation, Quality, Use, Access, and Trends
WHO: World Health Organisation
